# Brucellosis uveitis: A case report and literature review

**DOI:** 10.1097/MD.0000000000046416

**Published:** 2025-12-19

**Authors:** Jia Wei, Ru Chen, Tianlong Liu, Gengjun Jiao, Bin Zhang

**Affiliations:** a Department of Infectious Disease, The 940th Hospital Joint Logistics Support Forces of PLA, Lanzhou, China; b Department of Cardiology, The Second Affiliated Hospital of Lanzhou University, Lanzhou, China; c Department of Pharmacy, The 940th Hospital Joint Logistics Support Forces of PLA, Lanzhou, China.

**Keywords:** Brucellosis, complications, uveitis

## Abstract

**Rationale:**

Ocular involvement is an uncommon but serious complication of brucellosis, often leading to diagnostic delays and potential vision loss. This report presents a case of brucellosis uveitis to raise clinical awareness and discusses its diagnosis and management.

**Patient concerns:**

A 65-year-old female farmer presented with a 1-year history of intermittent fever and lower back pain, followed by progressive visual impairment and blurred vision over 2 months.

**Diagnoses:**

Based on the epidemiological history, serological tests that were positive for both the Rose Bengal plate test and the tube agglutination test, lumbar magnetic resonance imaging findings, and an ophthalmological examination, the patient was diagnosed with brucellosis accompanied by left ocular uveitis and optic nerve atrophy after other causes were excluded.

**Interventions:**

The patient was treated with a systemic triple-antibiotic regimen (doxycycline, rifampicin, and compound sulfamethoxazole) for 3 months, alongside topical anti-inflammatory and antibiotic-steroid eye drops.

**Outcomes:**

After 2 months of treatment, the uveitis had resolved significantly, and visual acuity was restored to 1.0 in both eyes. The patient remained asymptomatic at 1-year follow-up.

**Lessons:**

This case underscores the importance of considering brucellosis in the differential diagnosis of uveitis, especially in endemic areas. A combination of serology, imaging, and specialist examination is crucial for timely diagnosis and prevention of irreversible ocular damage.

## 1. Introduction

Brucellosis is a pervasive zoonotic disease caused by Brucella species, presenting with nonspecific systemic symptoms such as fever, arthralgia, and fatigue.^[1]^ While it can affect multiple organ systems, ocular complications are relatively rare and poorly characterized in the literature.^[2,3]^ Among these, uveitis is one of the most sight-threatening manifestations, which may present as anterior, intermediate, posterior, or panuveitis.^[4]^ The nonspecific nature of ocular symptoms, which often manifest only in the chronic phase of brucellosis and mimic other etiologies, poses a significant diagnostic challenge, frequently leading to delays.^[2]^ This report details a case of brucellosis-related uveitis and reviews the literature to highlight the clinical features, diagnostic strategies, and management approaches for this serious complication, aiming to improve early recognition and treatment outcomes.

This study was approved by the Medical Ethics Committee of the 940th Hospital Joint Logistics Support Forces of PLA and followed the tenets of the Declaration of Helsinki. The patient signed a written informed consent form after an explanation of the nature and possible consequences of the study.

## 2. Case summary

A 65-year-old female farmer was admitted to the hospital on August 3, 2023, due to a 1-year history of intermittent fever and lower back pain, accompanied by progressive visual impairment and blurred vision for 2 months. Her symptoms began in August 2022 with an intermittent, unexplained fever (up to 37.8℃), chills, lower back pain, fatigue, and night sweats, for which she did not seek medical care. Her visual symptoms developed in June 2023. She reported a history of direct contact with livestock (cattle and sheep).

**Figure 1.**
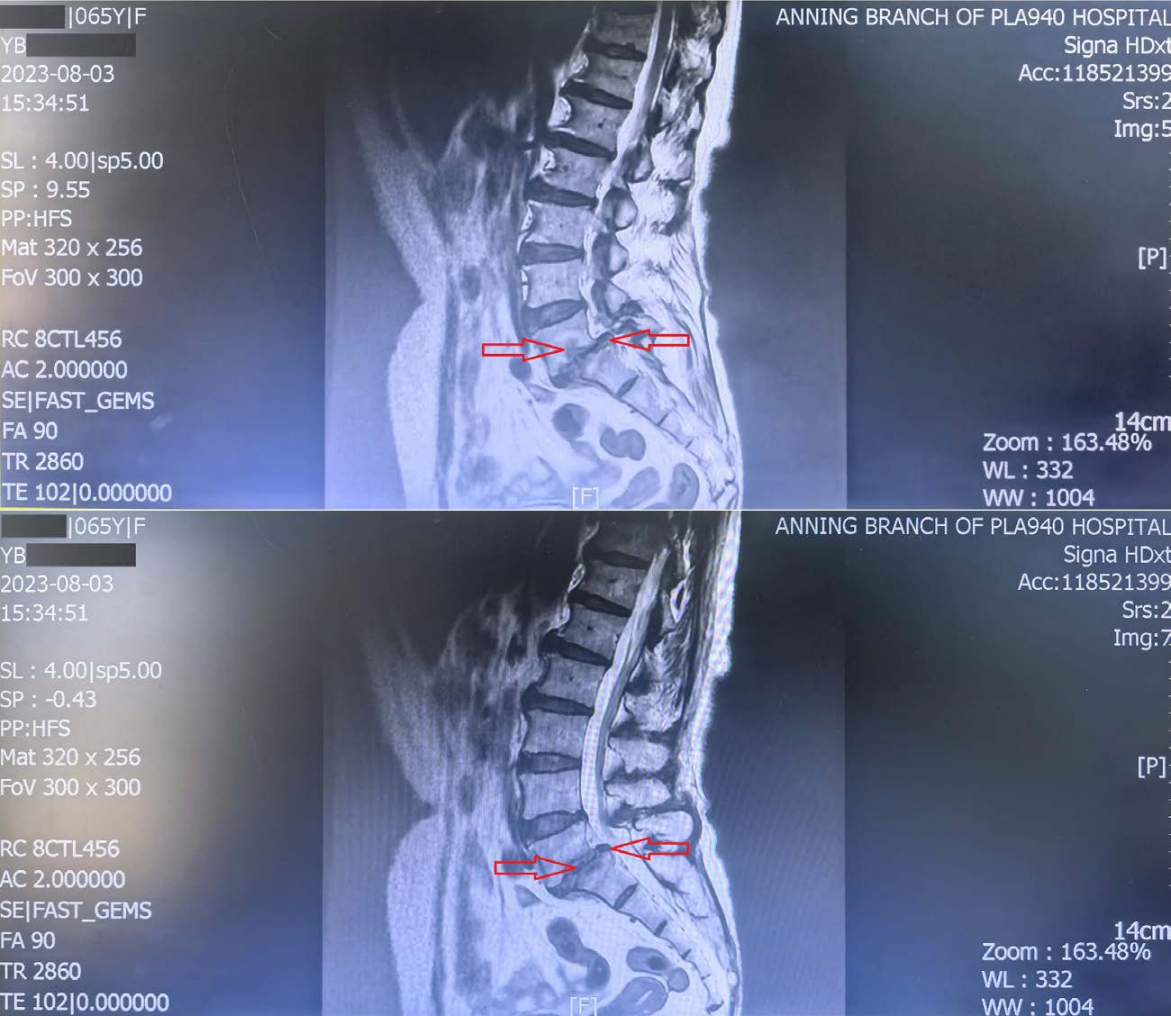
Magnetic resonance image of the patient’s lumbar spine. The red arrow indicates the site of the lesion: the lumbar magnetic resonance imaging showed abnormal signals in the body of L5 and S1 vertebrae and the L5-S1 intervertebral space, and slight effusion in the subcutaneous soft tissue of the lumbar and sacral region. L5 = fifth lumbar vertebra, S1 = the first sacral vertebra.

Physical examination revealed lumbar tenderness and mild limitation of lumbar spine movement. Serological tests were positive (Rose Bengal plate test and serum tube agglutination test with an antibody titer of 1:100++). Blood tests, liver and kidney function tests, cardiac enzyme tests, tuberculin test, fluorescent treponemal antibody absorption test, rheumatoid factor, antinuclear antibody test, and toxoplasmosis immunoglobulin m and immunoglobulin g antibodies showed no significant abnormalities. Lumbar magnetic resonance imaging showed abnormal signals in the fifth lumbar vertebra and the first sacral vertebra vertebral bodies and the fifth lumbar vertebra–the first sacral vertebra intervertebral space, along with slight effusion in the subcutaneous soft tissue of the lumbosacral region (Fig. 1).

**Figure 2.**
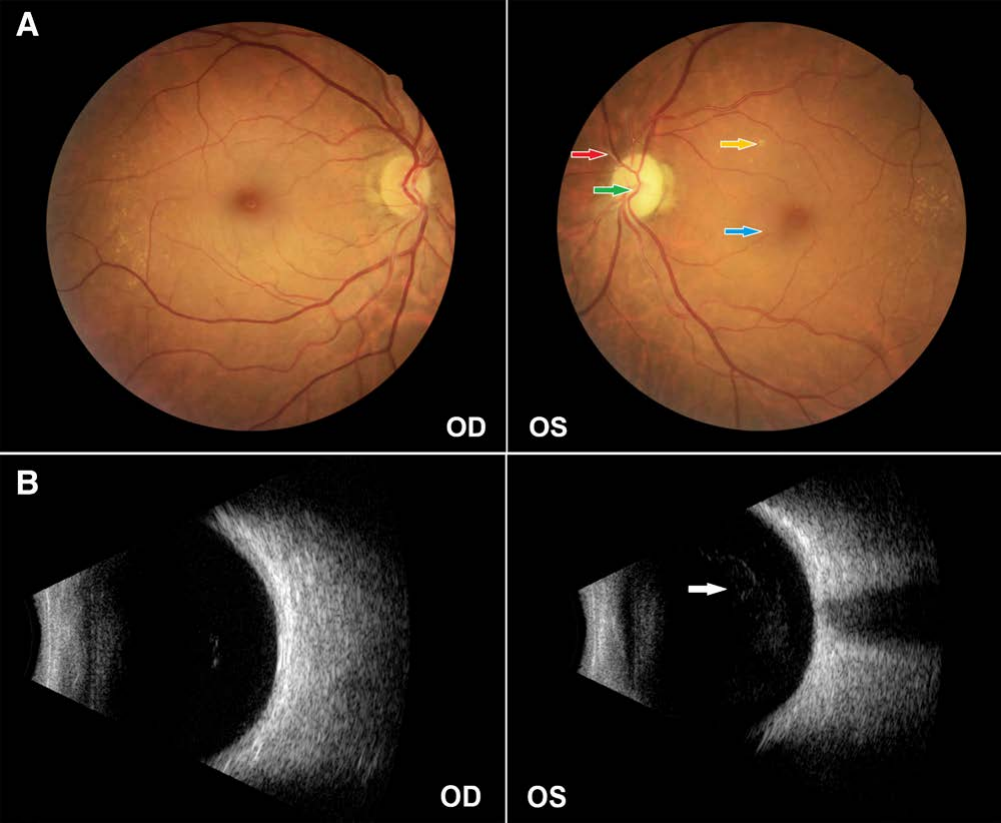
Ocular examination findings at diagnosis. (A) Fundus photographs of the right eye (OD) and left eye (OS). OD: unremarkable. OS: shows optic disc pallor (green arrow), arterial light reflex enhancement, sheathing along the superonasal branch retinal artery (red arrow), and arteriovenous crossing indentations. Multiple yellowish-white exudates are present throughout the retina (yellow arrow). Note retinal edema at the posterior pole and poor foveal reflex (blue arrow). (B) Ocular B-scan ultrasonography of both eyes. OD: within normal limits. OS: shows vitreous opacities (white arrow), suggestive of inflammatory exudates, and features consistent with optic nerve atrophy. OD = oculus dexter (right eye), OS = oculus sinister (left eye).

Ophthalmological examination revealed a visual acuity of 1.0 in the right eye and 0.08 in the left eye. Slit-lamp fundus examination of the right eye showed a clear optic disc, no retinal hemorrhage, and a visible fovea reflex in the macular area. The left eye had a pale optic disc, no retinal hemorrhage, and an unclear fovea reflex. The fundus photography of the left eye shows a clear optic disc boundary, pale color, strong arterial reflection, a white sheath can be seen in the superior nasal branch arteries, and a cross indentation of arteries and veins (+). Yellowish-white exudation can be seen in the retina, posterior pole retinal edema, and unclear reflection in the fovea centralis of the macula (Fig. 2A). Ocular ultrasonography revealed vitreous opacification in the left eye, suggestive of inflammatory exudates, and optic nerve atrophy (Fig. 2B).

The final diagnoses were: brucellosis, left ocular brucellosis uveitis, and left optic nerve atrophy.

**Figure 3.**
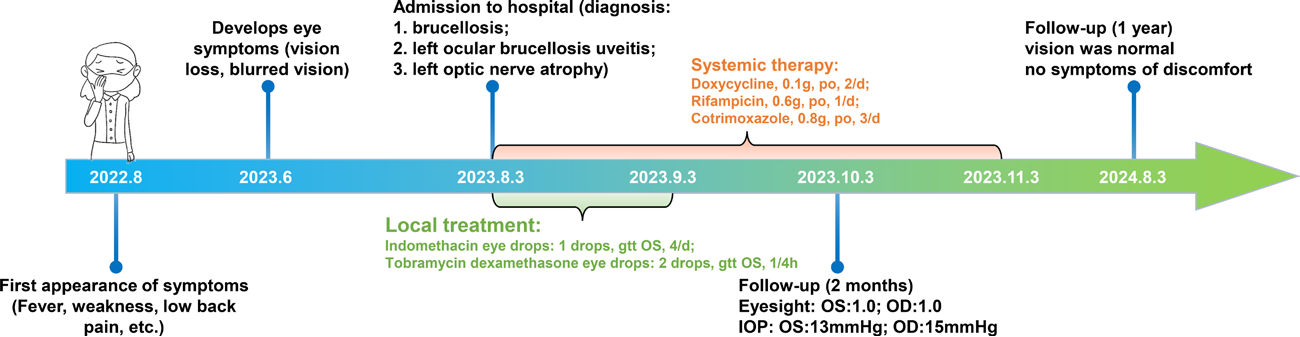
Clinical duration and treatment of brucellosis uveitis. This figure provides an overview of the diagnosis, treatment, and outcomes of patients with Brucellosis uveitis. The initial systemic symptoms (such as fever and weakness) are followed by ocular manifestations. The patient was diagnosed with brucellosis complicated with uveitis of the left eye and optic nerve atrophy. Systemic treatment includes doxycycline, rifampicin compound sulfamethoxazole, plus local indomethacin, and tobramycin/dexamethasone eye drops. Vision and intraocular pressure returned to normal within 2 months of follow-up, and symptoms were completely relieved within 1 year. (OS = left eye, OD = right eye; IOP = intraocular pressure).

Treatment: according to the “Diagnosis and Treatment Guidelines for Brucellosis of the Ministry of Health (2012),” “Provisional,” “Diagnostic Criteria for brucellosis of the People’s Republic of China (WS269-2019),” the 9th edition of the textbook “Infectious Diseases” published by People’s Medical Publishing House, and the recommended treatment regimens for brucellosis complications in a review titled “Human brucellosis” published in The Lancet Infectious Diseases in 2007, patients with complications of brucellosis are treated with doxycycline 0.1 g orally 2/day (3 months), rifampicin 0.6 g orally 1/day (3 months), and compound sulfamethoxazole 0.8 g orally 2/day (3 months). Topical ocular therapy included 1 drop of indomethacin eye drops 4 times daily for 1 month and 2 drops of Tobramycin and Dexamethasone eye drops every 4 hours for 1 month. Timeline of the patient’s diagnosis and treatment process is shown in Figure 3.

Follow-up: After 2 weeks of treatment, the patient’s vision and symptoms had improved. Weekly monitoring of liver and kidney function and blood counts during treatment showed no abnormalities, and no adverse drug reactions were observed. After 2 months, visual acuity was 1.0 in both eyes, with intraocular pressures of 15 (right) and 13 mm Hg (left). A 1-year telephone follow-up confirmed normal vision and no recurrence of symptoms.

## 3. Discussion

Brucellosis is a systemic disease with diverse clinical manifestations, and diagnosis is usually based on clinical indicators, serological tests, or culture trials.^[1]^ However, when the disease affects delicate organs such as the eyes, the diagnostic process becomes more challenging, frequently resulting in misdiagnosis or delay.^[5]^ Brucellosis uveitis is divided into 4 types based on the anatomical location of inflammation: anterior, intermediate, posterior, and panuveitis. Sungur et al reported that 21% of 132 confirmed brucellosis patients had ocular involvement, with anterior uveitis (41%) and choroiditis (32%) being the most common, followed by panuveitis, papillitis, and retinal hemorrhage (9% each). Notably, all patients with anterior uveitis were in the acute stage of brucellosis, while other ocular manifestations were detected in the chronic stage.^[6]^ A large study in Peru by Rolando et al found a 3.4% prevalence of ocular involvement among 1551 brucellosis patients over 26 years, with posterior uveitis (35%) and panuveitis (32%) being the most frequent manifestations.^[4]^ A meta-analysis of 27 studies including 159 patients with ocular brucellosis reported a pooled prevalence of 52.2% for ocular involvement and 17.6% for conjunctivitis. Among these patients, 27.7% had systemic infection symptoms, and many experienced vision impairment or loss. Only 37.1% of treated patients regained their vision.^[7]^

Brucellosis can cause a spectrum of ocular lesions, including lacrimal glanditis, conjunctivitis, periorbital cellulitis,^[3]^ and in severe cases, panuveitis and vitritis. Associated symptoms include blurred vision, ocular pain, excessive tearing, visual distortion, foreign body sensation, cotton wool spots, vitreous hemorrhage, and retinal exudative detachment, all of which can seriously threaten vision.^[5]^ The etiology of uveitis is multifactorial, often involving environmental, cultural, and genetic factors.^[8,9]^ Brucellosis-induced uveitis can present with granulomatous or non-granulomatous features and may be unilateral or bilateral.^[10]^ Some patients may also develop neuro-ophthalmic complications such as optic disc edema, retrobulbar neuritis, and optic neuritis.^[11]^ Other reported manifestations include endophthalmitis, keratitis, and cranial nerve palsy.^[12,13]^ Ocular symptoms typically appear in the chronic stage of systemic infection, with early-stage detection being relatively rare.^[14]^ Many studies identify uveitis, particularly posterior uveitis, as the most common form of ocular involvement,^[15]^ although some reports suggest that conjunctivitis is more frequent.^[16]^

The prognosis for brucellosis-related posterior uveitis or panuveitis is often poor. Ocular brucellosis can lead to vision impairment or blindness, as well as complications such as cataract, glaucoma, macular degeneration, vitreous changes, retinal vascular proliferation, and detachment, further complicating management.^[17]^

The diagnosis of brucellosis uveitis relies on clinical ophthalmological examination combined with laboratory tests, including the standard agglutination test, Coombs test, 2-mercaptoethanol test, and blood or bone marrow culture.^[18]^ In some cases, aqueous humor culture, serological testing of ocular fluids, or biopsy may be necessary.^[19]^ The emergence of metagenomic next-generation sequencing has improved diagnostic capabilities by allowing unbiased, high-throughput sequencing of all microbial deoxyribonucleic acid/ribonucleic acid in a sample, enabling rapid pathogen identification.^[20]^ For brucellosis uveitis, metagenomic next-generation sequencing testing of aqueous humor can provide a definitive diagnosis quickly.^[21]^ In this case, we quickly obtained a reliable diagnosis based on the patient’s epidemiological history of contact with cattle and sheep, clinical symptoms such as fever, night sweats, weakness, and joint pain, as well as a positive Rose Bengal plate test and a 1:100 serum tube agglutination test, enabling the patient to start standardized treatment as soon as possible.^[22]^ This case illustrates the practical value of serological testing in the first-line clinical diagnosis of brucellosis. Although bone marrow or blood culture is regarded as the gold standard for etiological diagnosis and can be used for strain identification, due to its long culture period, high biosafety requirements, and the fact that a clear diagnosis has been obtained through serology in this case, no invasive procedures were performed on the patient to obtain culture samples.^[23]^

Differential diagnoses including tuberculosis, syphilis, rheumatological diseases, and toxoplasmosis were ruled out through negative tuberculin test, fluorescent treponemal antibody-absorption, rheumatoid factor, antinuclear antibody, and toxoplasmosis serology, as well as a normal chest X-ray. The positive anti-Brucella serology confirmed the diagnosis and excluded other causes of uveitis. This case highlights a common diagnostic challenge in clinical practice. The clinical presentation of brucellosis uveitis often mimics other infectious or noninfectious intraocular inflammations, such as tuberculous uveitis, syphilitic uveitis, sarcoidosis, toxoplasmosis, and Behçet disease, which may coexist or be misdiagnosed in endemic areas, leading to delays.^[24,25]^ For instance, tuberculous uveitis can also present as posterior uveitis or panuveitis with retinal vasculitis or choroidal nodules,^[26]^ while sarcoidosis is frequently associated with vitreous “snowball” opacities, eriphlebitis, and iris nodules.^[27]^ Therefore, a comprehensive approach, integrating detailed epidemiological history, review of systemic symptoms, serological tests, and imaging, is essential. In refractory or atypical cases, analysis of intraocular fluids or tissue biopsy may be required to exclude other etiologies.^[28]^ The patient had symptoms for 1 year prior to diagnosis, but their nonspecific nature led to a delay, resulting in ocular involvement. Given the poor prognosis of undiagnosed ocular brucellosis, we emphasize the need for enhanced public education and awareness in endemic areas to improve prevention.

The optimal antimicrobial strategy for brucellosis, especially when complicated by ocular involvement, aims to ensure penetration into privileged sites and prevent relapse. In an international context, the World Health Organization recommends 2 primary regimens: an oral combination of doxycycline and rifampicin for a minimum of 6 weeks, or a regimen where rifampicin is replaced by streptomycin for the first 2 to 3 weeks.^[29]^ The triple oral regimen (doxycycline, rifampicin, and sulfamethoxazole-trimethoprim) utilized in this case, while differing from the World Health Organization’s dual-therapy suggestions was selected for several reasons. This approach aligns with Chinese guidelines for complicated brucellosis and was chosen for its practical advantages in an outpatient setting, avoiding the need for prolonged parenteral administration of aminoglycosides and their associated toxicity monitoring. Furthermore, this choice is supported by evidence discussed in the same authoritative review,^[29]^ which notes concerns regarding treatment failure and relapse with standard regimens and explores the potential benefit of triple-drug combinations.

Current treatment guidelines for brucellosis recommend combination antibiotic therapy, typically including at least 2 antibiotics such as doxycycline, rifampicin, streptomycin, gentamicin, or trimethoprim-sulfamethoxazole,^[29]^ particularly for ocular involvement, where a combination of systemic and topical steroids for 2 to 4 weeks is beneficial.^[10]^ A comprehensive review highlighted the superiority of triple-drug regimens over dual therapy, with monotherapy being inferior due to high failure rates. It also demonstrated that treatment durations of 6 weeks or longer were significantly more effective than shorter courses.^[15]^

For this patient, we implemented an evidence-based treatment strategy for brucellosis uveitis, consisting of a triple oral antibiotic regimen (doxycycline, rifampicin, and compound sulfamethoxazole) for 3 months to eradicate the infection and prevent relapse. Ocular inflammation was managed with topical indomethacin and tobramycin-dexamethasone eye drops. Vision improved within 2 weeks of starting treatment. Topical drops were discontinued after 1 month, while systemic therapy was completed over 3 months. The patient showed excellent compliance. At the 2-month follow-up, uveitis had resolved, and best-corrected visual acuity was 1.0 in both eyes, with normal intraocular pressures. At 1 year, the patient remained asymptomatic with no signs of active disease or recurrence. This case demonstrates the efficacy of a prolonged triple-drug regimen for treating brucellosis uveitis, which led to a complete resolution of inflammation and a restoration of visual function.

Despite the valuable insights provided by this case report and literature review, several limitations should be acknowledged. First, the existing epidemiological data on ocular involvement in brucellosis exhibit significant heterogeneity, with reported prevalence rates varying widely across studies. This variability likely stems from differences in study populations, diagnostic criteria, and healthcare-seeking behaviors, making it challenging to define a precise disease burden. Second, the available literature is characterized by a geographic bias, with most studies originating from endemic regions, which may limit the generalizability of the findings to non-endemic areas. Furthermore, as ocular complications are relatively rare, the current evidence is largely based on case reports and small case series, inherently limiting the statistical power and the strength of conclusions regarding optimal management and prognosis. Finally, as a single case report, our study has inherent constraints. While the diagnosis was strongly supported by serological and clinical evidence, the inability to obtain bacteriological confirmation through culture remains a limitation, albeit a common one in clinical practice due to the fastidious nature of Brucella.^[23]^ Future large-scale, multicenter prospective studies are warranted to better define the clinical spectrum and establish evidence-based treatment guidelines for this serious complication.

In summary, brucellosis can lead to severe systemic and ocular complications. The nonspecific nature of ocular manifestations makes them prone to being missed or misdiagnosed. This case highlights the importance of considering brucellosis in the differential diagnosis of uveitis in endemic areas. To improve patient outcomes, we emphasize 2 critical strategies: first, prompt initiation of multidisciplinary management, involving infectious disease specialists and ophthalmologists from the outset to guide simultaneous systemic and ocular treatment; and second, the development of region-specific diagnostic protocols in endemic areas that include routine Brucella serological testing for patients with unexplained uveitis. Such measures are essential for timely diagnosis and intervention, minimizing the risk of irreversible vision loss.

## Acknowledgments

We are also grateful to all the researchers, including the physicians, nurses, and technicians, who participated in this study.

## Author contributions

**Conceptualization:** Bin Zhang.

**Data curation:** Ru Chen.

**Funding acquisition:** Tianlong Liu.

**Resources:** Ru Chen.

**Supervision:** Gengjun Jiao.

**Writing – original draft:** Jia Wei.

**Writing – review & editing:** Bin Zhang.
